# Cytosolic N-terminal formyl-methionine deformylation derives cancer stem cell features and tumor progression

**DOI:** 10.1038/s41598-024-65701-1

**Published:** 2024-06-28

**Authors:** Dasom Kim, Jongeun Lee, Ok-Hee Seok, Yoontae Lee, Cheol-Sang Hwang

**Affiliations:** 1 Department of Life Sciences, Pohang University of Science and Technology, Pohang, Gyeongbuk 37673 Republic of Korea; 2 Department of Life Sciences, Korea University, Seoul, 02841 Republic of Korea

**Keywords:** Biochemistry, Cancer, Cell biology, Molecular biology, Stem cells

## Abstract

Eukaryotic cells can synthesize formyl-methionine (fMet)-containing proteins not only in mitochondria but also in the cytosol to some extent. Our previous study revealed substantial upregulation of N-terminal (Nt)-fMet-containing proteins in the cytosol of SW480 colorectal cancer cells. However, the functional and pathophysiological implications remain unclear. Here, we demonstrated that removal of the Nt-formyl moiety of Nt-fMet-containing proteins (via expressing *Escherichia coli* PDF peptide deformylase) resulted in a dramatic increase in the proliferation of SW480 colorectal cancer cells. This proliferation coincided with the acquisition of cancer stem cell features, including reduced cell size, enhanced self-renewal capacity, and elevated levels of the cancer stem cell surface marker CD24 and pluripotent transcription factor SOX2. Furthermore, deformylation of Nt-fMet-containing proteins promoted the tumorigenicity of SW480 colorectal cancer cells in an in vivo xenograft mouse model. Taken together, these findings suggest that cytosolic deformylation has a tumor-enhancing effect, highlighting its therapeutic potential for cancer treatment.

## Introduction

Colorectal cancer (CRC) ranks third in global cancer incidence, accounting for approximately 10% of all cancer cases, and is the second leading cause of cancer-related deaths^1^. Its development is influenced by a complex interplay of risk factors, including age, diet, environmental exposure, family history, and personal factors such as polyp presence^2^. Despite significant advancements in CRC diagnosis and treatment, recurrence and mortality rates remain high. This is partly owing to the presence of cancer stem cells (CSCs), which have remarkable self-renewal abilities and are critical for tumorigenesis by affecting CRC initiation, progression, metastasis, and recurrence^3^. Cancer stem cells constitute a small fraction of the tumor mass (0.01–2%), and can emerge from non-CSCs through de-differentiation, adding phenotypic plasticity and fueling further tumor development^4^.

In bacteria and bacteria-descended eukaryotic organelles, including mitochondria and plastids, protein synthesis begins with N-terminal (Nt)-formyl-methionine (fMet)^5,6^. Formyltransferase (FMT) catalyzes the attachment of the formyl moiety from 10-formyl tetrahydrofolate (10-fTHF) to initiator Met-tRNAi, resulting in Nt-formylated Met-tRNAi (fMet-tRNAi)^5,6^. Subsequently, ribosomes initiate protein synthesis via fMet-tRNAi. When Nt-fMet-containing polypeptides emerge from a ribosomal exit tunnel, ribosome-associated peptide deformylase (PDF) removes the Nt-formyl moiety from the nascent polypeptides, generating unmodified Met at the N-termini^7^. Thus, the population of fMet-containing proteins can be controlled by the counteractions of FMT and PDF.

The apparent absence of FMT, fMet-tRNAi, and fMet-containing proteins in the cytosol of eukaryotes where protein synthesis mainly occurs using unformylated Met-tRNAi suggests that the production of Nt-formylated proteins is confined to bacteria and bacteria-derived eukaryotic organelles^5^. However, recent studies have challenged this assumption by revealing the presence of nuclear DNA-encoded fMet-containing proteins in the cytosol of yeast and human cells^6,8–10^.

In addition to its role in the initiation of protein synthesis, Nt-fMet can function as a specific protein degradation signal in cellular proteins termed fMet/N-degron, targeted by the eukaryotic fMet/N-degron pathway^8^. Notably, the downregulation of cytosolic Nt-formylated proteins in yeast through the expression of *Escherichia coli* PDF (*Ec*PDF) deformylase increases cellular susceptibility to undernutrition and cold stress, underscoring the physiological importance of cytosolic Nt-formylation^8^. Moreover, cytosolic Nt-formylated proteins are upregulated in several cancer cell lines, including SW480 colorectal cancer cells^9^. However, the pathophysiological roles of cytosolic Nt-formylation in these cancer cells remain unclear.

In this study, we show that cytosolic deformylation of Nt-formylated proteins enhances the proliferation of SW480 colorectal cancer cells and promotes cancer stem cell (CSC) properties and in vivo tumorigenesis in a xenograft model. Furthermore, our comparative analysis of The Cancer Genome Atlas (TCGA) database reveals a significant upregulation of the mRNA levels of *Homo sapiens* PDF (*Hs*PDF) in tissues from colorectal cancer patients.

## Materials and methods

### Antibodies

The following primary antibodies were used: anti-flag (F3165; Sigma Aldrich, MO, USA), anti-tubulin (T5168; Sigma Aldrich), anti-TOM20 (sc-17764; Santa Cruz, CA, USA), and anti-SOX2 (ab97959; Abcam, CB, UK). The anti-fMet antibody was purified as previously described with slight modifications^9^. Briefly, anti-fMet sera were negatively selected using Affi-gel-10/15 resins (1536098; Bio-Rad, CA, USA) conjugated with extracts from *Ec*PDF_3f_-1 and *Ec*PDF_3f_-2 SW480 cells. After three rounds of negative selection with the resulting resins, the unbound sera were diluted in PBS-T (phosphate-buffered saline with 0.05% Tween 20, pH 7.4) at a 1:500 ratio and pre-incubated with polyvinylidene fluoride (PVDF; IPVH0010; Merck, NJ, USA), on which extracts from *Ec*PDF_3f_-1 SW480 cells were electro-transferred before use. The secondary antibodies were goat anti-rabbit IgG (170–6515; Bio-Rad) and anti-mouse IgG (170–6516; Bio-Rad).

### Plasmid construction

The plasmids and primers used in this study are listed in Supplementary Tables 1 and 2. To construct pCH5542, *EcPDF* DNA was PCR-amplified from pCH5540 using the primer pair OCH5578/5579. Simultaneously, *3*× *FLAG* DNA was PCR-amplified using the primer pair OCH5576/5577. Subsequently, both *EcPDF* and *3*× *FLAG* PCR products were used as templates for overlap extension PCR using the primer pair OCH5578/OCH5577. The resulting PCR product was digested with *Kpn*I/*Xho*I and subsequently ligated into *Kpn*I/*Xho*I-digested pcDNA3(+).

### Cell culture and transfection

SW480 cells were cultured in RPMI-1640 (11875-093; Cytiva, MA, USA) supplemented with 10% fetal bovine serum (FBS; SH30919.03; Cytiva) and transfected with either pcDNA3 or pCH5542 (pcDNA3-*Ec*PDF_3f_) using Lipofectamine 2000 (11668019; Thermo Fisher Scientific, MA, USA) for 24 h. The cells were subsequently treated with 1–2 mg/ml G418 (G-1033; AG Scientific, CA, USA) for two weeks. The cell medium was replaced with fresh medium supplemented with G418 every 3–4 days. Colonies were picked using cloning cylinders and cultured in RPMI-1640 supplemented with 10% FBS. *Ec*PDF expression in *Ec*PDF_3f_ stable cells was confirmed by immunoblotting using an anti-flag antibody.

HT29 cells were cultured in Dulbecco’s modified Eagle’s medium (DMEM) (SH30243.01; Cytiva) containing 10% FBS. For cell proliferation assays and flow cytometry, HT29 cells were seeded in a 12-well culture plate and transiently transfected with either pcDNA3 or pCH5542 (pcDNA3-*Ec*PDF_3f_) (1.5 µg) using 7.5 µl Lipofectamine LTX (15338030; Thermo Fisher Scientific) for 1–2 days before analysis. For immunoblotting, HT29 cells were seeded in a 12-well culture plate and transiently transfected with either OGS411 or pCH5540 (OGS411-*Ec*PDF_3f_) (1 µg) using 4 μl polyethylenimine solution (1 mg/ml) for 3 days. Additional information on the cell lines used in this study is provided in Supplementary Table 3.

### Subcellular fractionation

Stably *Ec*PDF_3f_-expressing SW480 cells were fractionated using a Qproteome Mitochondria Isolation Kit (37612; Qiagen, Hilden, Germany). Subsequently, 2.5 μg of the mitochondrial and cytosolic fractions were subjected to immunoblotting with the indicated antibodies.

### Immunoblotting analysis

Harvested cells were lysed with RIPA buffer (89900; Thermo Fisher Scientific) supplemented with protease inhibitor cocktail (4693132001; Sigma Aldrich) and 20 µg/ml actinonin (HY-113952; MedChemExpress, NJ, USA). The cell lysates were incubated on ice for 20 min, briefly sonicated, and centrifuged at 12,000×*g* for 10 min at 4 °C. The collected supernatants were diluted to 2 μg/μl, mixed with 4× SDS sample buffer at a 3:1 ratio, and heated at 70 °C for 10 min. Subsequently, 10 μl of each sample was subjected to immunoblotting with the specified antibodies. For immunoblotting with multiple antibodies, the membrane was cut into several pieces before incubation with specific antibodies. Immunoblots were developed with ECL substrates in chemiluminescence mode using Amersham Imager AI680 (GE HealthCare, IL, USA). Immunoblotting band intensities were quantified using ImageJ software (https://imagej.nih.gov/ij/). The uncropped immunoblotting images of the main figures are provided in Supplementary Information.

### Cell proliferation assay

Stably *Ec*PDF_3f_-expressing SW480 cells (1 × 10^5^) in RPMI-1640 containing 10% FBS were seeded into each well of a 24-well plate. Alternatively, transiently *Ec*PDF_3f_-expressing HT29 cells (2 × 10^5^) in DMEM containing 10% FBS were seeded into each well of a 12-well plate. On the specified day, the cells were harvested and counted using a TC20 automated cell counter (Bio-Rad).

### Soft agar colony formation assay

A 1% agarose solution and RPMI-1640 supplemented with 10% FBS were mixed in a 1:1 ratio, and 2 ml of the mixture was poured into each well of a 6-well plate. Once the bottom layer solidified, the melted 0.7% agarose solution and RPMI-1640 supplemented with 10% FBS containing 2.5 × 10^3^ cells were mixed in a 1:1 ratio, and 2 ml of the mixture was gently poured on top of the solidified bottom layer. The plates were incubated at 37 °C in a 5% CO_2_ incubator to allow the top layer to solidify. Throughout the colony formation period, the top layer was maintained in fresh medium every 2–3 days to ensure nutrient replenishment. After two weeks, the agar layers were stained with 0.5% crystal violet to visualize the colonies, and images were captured for analysis.

### Phalloidin staining

Cultured cells were fixed with 4% paraformaldehyde in PBS, permeabilized with 0.1% Triton X-100 in PBS, and stained with phalloidin conjugated to Alexa Fluor 594 (A12381; Thermo Fisher Scientific) for 30 min. The phalloidin staining solution (0.2 µM phalloidin and 1% bovine serum albumin [BSA] in PBS) was used. Fluorescence was observed using a Zeiss Axio Scope-A1. Cell sizes were measured using the ImageJ software.

### Spheroid assay

Non-confluent SW480 cells were trypsinized, dispersed into single-cell suspensions, and plated at a density of 500 cells per well in a 24-well ultra-low attachment plate (CLS3473; Corning, NY, USA) with DMEM/F-12 containing 10 ng/ml bFGF (F0291; Sigma-Aldrich) and 20 ng/ml EGF (AF-100–15; Thermo Fisher Scientific) for 10 days. The culture medium was replaced every 4 days. Spheres over 50 μm in size were enumerated using Olympus CKX53 microscope at × 20 magnification.

### RT-qPCR

Total RNA was isolated from control and *Ec*PDF_3f_ SW480 cells using the RNeasy Mini Kit (74104; Qiagen). cDNA was synthesized using the RevertAid First Strand cDNA Synthesis Kit (K1622; Thermo Fisher Scientific). RT-qPCR was performed using the SYBR Green Master Mix (4367659; Applied Biosystems, MA, USA) on a StepOnePlus Real-Time PCR System (Applied Biosystems). Relative mRNA levels of target genes were normalized to β-actin using the ΔΔCt method. Primer sequences are listed in Supplementary Table 4.

### Flow cytometry

Control vector- or *Ec*PDF_3f_-expressing SW480 and HT29 cells were harvested and resuspended in FACS buffer (PBS + 2% FBS) at 1 × 10^7^ cells/ml. Cell suspensions were incubated with anti-CD24-FITC (1:20 dilution; 11–0247-41; Thermo Fisher Scientific) or isotype control (11-4714-81; Thermo Fisher Scientific) on ice for 30 min. Subsequently, the cells were diluted in FACS buffer and analyzed using a FACSVerse flow cytometer (BD Biosciences, NJ, USA). A total of 25,000 events were acquired, and events with FSC-A values less than 50,000 were excluded from the analysis. The data were subsequently gated and plotted using FACSuite Software (BD Biosciences).

### In vivo xenograft assay

BALB/c nude mice (5 weeks old; male) were obtained from Orient Bio (Seongnam, Gyeonggi, South Korea) and acclimated for 1 week. The mice were fed standard rodent chow and water ad libitum and maintained in a specific pathogen-free animal facility with a standard 12-h light/12-h dark cycle. For the xenograft tumor growth assay, vector alone- and *Ec*PDF-expressing SW480 cells (5.0 × 10^6^ cells) in 50 µl culture medium were mixed with 50 µl Matrigel (CLS354234; Corning). The cell mixtures were subcutaneously injected into the posterior flank of 6-week-old male BALB/c nude mice. Tumor volume was measured every 2 days for a period of 26 days and calculated using the following formula: 1/2 × (largest diameter) × (smallest diameter)^2^. On day 26, tumor tissues were extracted and lysed in RIPA buffer supplemented with a protease inhibitor cocktail for immunoblotting analyses.

### TCGA database analysis

Gene expression data for normal and cancer cells (normalized RNA-seq FPKM-UQ) from patients with CRC were acquired from the TCGA database (TCGA-COAD, July 2023). The dataset comprises 41 normal and 473 cancer samples. Tumor stages were defined using the latest version of the American Joint Committee on cancer code at the time of diagnosis. Major tumor stages (I, II, III, or IV) were investigated for differences in gene expression. After normalization, the expression levels of *Hs*PDF gene were compared. Statistical analyses and visualization were performed using GraphPad Prism 9 (GraphPad Software, CA, USA).

### Statistical analysis

*P* values were determined using a two-tailed unpaired t-test and two-way analysis of variance (ANOVA) using GraphPad Prism 9/10. Statistical significance was set at *P* < 0.05.

## Results

### EcPDF expression increases the growth of colorectal cancer cells

**Figure 1:**
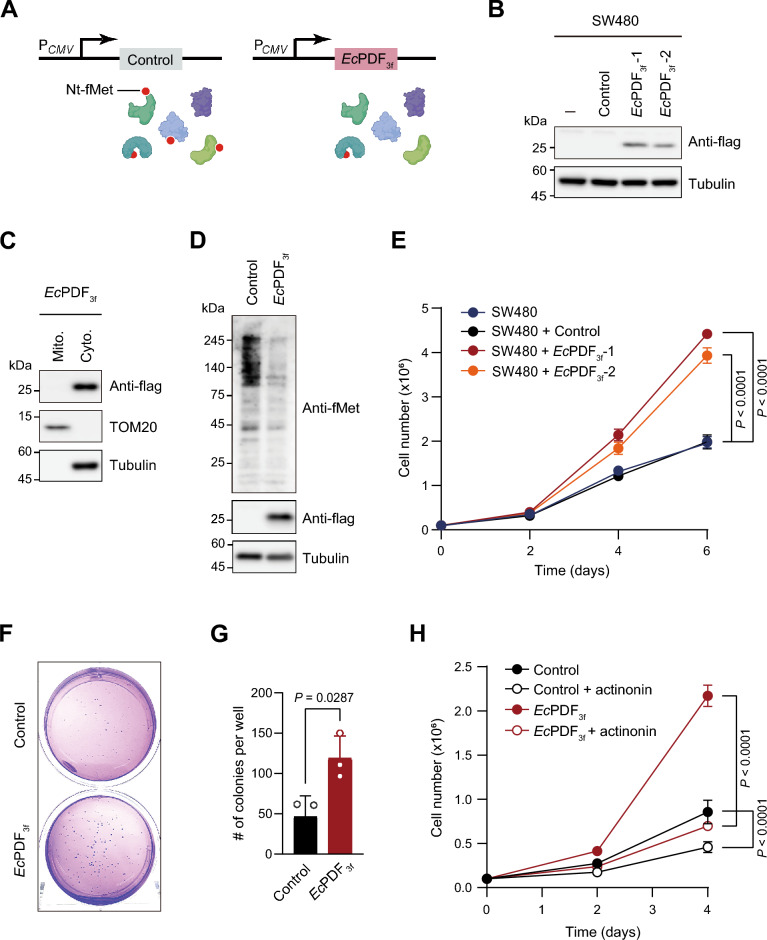
*Ec*PDF expression accelerates the growth of SW480 colorectal cancer cells. (**A**) Schematic depicting the expression of vector alone (control) or *Ec*PDF_3f_ under P_*CMV*_ promoter in SW480 cells. (**B**) Immunoblotting of *Ec*PDF_3f_ in mock, control (vector alone), *Ec*PDF_3f_-1, and *Ec*PDF_3f_-2 SW480 cells. Tubulin was hereafter used as the loading control. (**C**) Subcellular fractions of *Ec*PDF_3f_ in *Ec*PDF_3f_ SW480 cells, with TOM20 and tubulin serving as markers for the mitochondrial and cytosolic fractions, respectively. (**D**) Representative immunoblotting of Nt-fMet-containing proteins in control and *Ec*PDF_3f_ SW480 cells. (**E**) The growth of mock, control (vector alone), *Ec*PDF_3f_-1, or *Ec*PDF_3f_-2 SW480 cells. Data in the graph are presented as the mean ± SD of four replicate experiments; two-way ANOVA; *P* values are indicated in the figure. (**F**) Images of control and *Ec*PDF_3f_ SW480 colonies cultured on plates for 2 weeks. (**G**) Quantification of the number of colonies formed in (**F**). Error bars represent the mean ± SD of three replicate experiments; two-tailed *t*-test; *P* value is indicated in the figure. (**H**) The growth of vector alone and *Ec*PDF_3f_ SW480 cells in the absence or presence of actinonin (5 µg/ml final concentration). Data in the graph are presented as the mean ± SD of four replicate experiments; two-way ANOVA; *P* values are indicated in the figure.

We established stable SW480 cell lines carrying either a vector alone (control) or a C-terminally triple flag-tagged *Ec*PDF (*Ec*PDF_3f_) under the constitutive cytomegalovirus promoter (P_*CMV*_) (Fig. 1A, Supplementary Table 3). Given *Hs*PDF predominantly localizes to mitochondria, we utilized *Ec*PDF (lacking a mitochondria-targeting presequence) for cytosolic Nt-deformylation. *Ec*PDF_3f_ expression was confirmed in two independent *Ec*PDF_3f_-1 and *Ec*PDF_3f_-2 SW480 cell lines, but not in the control cell line by immunoblotting with an anti-flag antibody (Fig. 1B). Throughout this study, most experiments were performed using the *Ec*PDF_3f_-1 cell line, which is henceforth referred to as *Ec*PDF_3f_ cells.

Subcellular fractionation analyses showed that *Ec*PDF_3f_ was primarily localized in the cytosol owing to the lack of mitochondrial targeting presequence (Fig. 1C). As expected, immunoblotting with anti-fMet (which detects Nt-fMet-bearing proteins irrespective of the downstream sequences of Nt-fMet^9^) in *Ec*PDF_3f_ cells showed notably reduced levels of Nt-formylated proteins compared to those in control cells (Fig. 1D). The residual Nt-fMet-containing proteins detected in *Ec*PDF_3f_ cells might originate from mitochondrial fMet-containing proteins or incomplete deformylation of cytosolic proteins by *Ec*PDF.

Interestingly, *Ec*PDF_3f_-1 and *Ec*PDF_3f_-2 SW480 cells exhibited accelerated growth compared with control cells (Fig. 1E). Specifically, the doubling times for *Ec*PDF_3f_-1 and *Ec*PDF_3f_-2 cells were estimated to be ~ 26 h and ~ 27 h, respectively, which were shorter than the doubling time of the control cells (~ 33 h). The colony forming assay revealed a ~ 2.6-fold increase in colony number of *Ec*PDF_3f_ cells compared with control cells (Fig. 1F,G).

The increased growth of *Ec*PDF_3f_ cells was dramatically inhibited by actinonin, a PDF inhibitor (Fig. 1H). In this setting, the slight growth retardation of control cells by actinonin also indicates the involvement of endogenous human deformylase *Hs*PDF in addition to exogenous *Ec*PDF on cancer cell growth, as previously reported^11,12^. However, the more dramatic growth reduction observed in *Ec*PDF-expressing cells in the presence of actinonin suggests that cytosolic deformylation of Nt-formylated proteins is crucial for enhancing growth in SW480 cells upon *Ec*PDF expression.

### *Ec*PDF expression induces CSC properties

**Figure 2:**
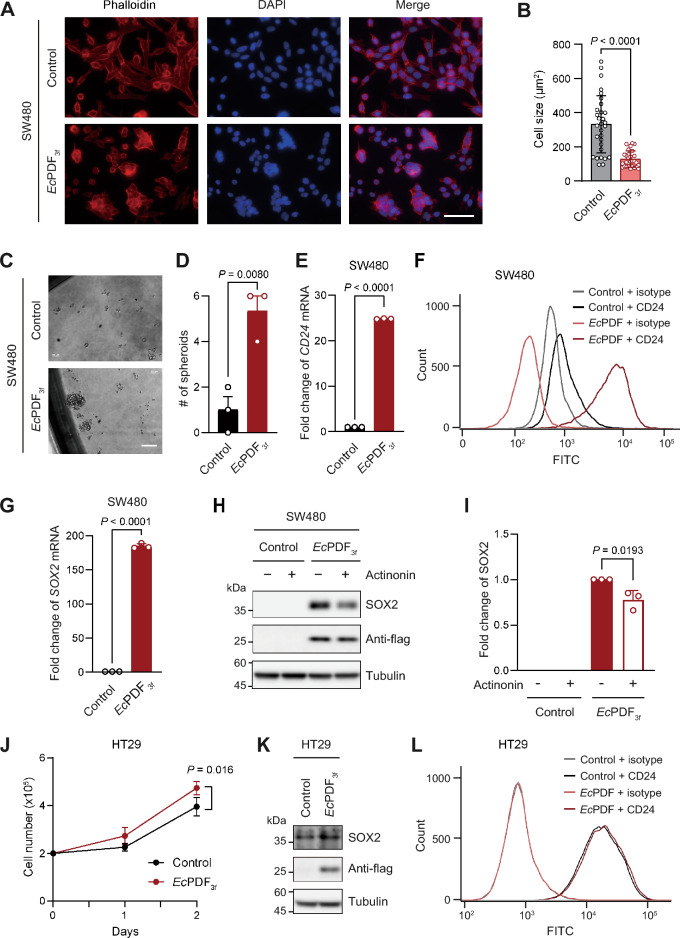
*Ec*PDF expression induces CSC-like phenotypes in colorectal cancer cells. (**A**) Morphologies of control and *Ec*PDF_3f_ SW480 cells. F-actin and nuclei were stained using Alexa Fluor 594-conjugated phalloidin and DAPI, respectively. Scale bars, 50 μm. (**B**) Quantification of cell sizes in (**A**). (**C**) Spheroid analyses of control and *Ec*PDF_3f_ SW480 cells. Spheroids with a diameter exceeding 50 μm were counted. Scale bars, 100 μm. (**D**) Quantification of data in (**C**). (**E**) The levels of *CD24* mRNA in control and *Ec*PDF_3f_ SW480 cells. (**F**) A representative FACS histogram depicting non-specific (isotype) and specific CD24 fluorescence patterns in control and *Ec*PDF_3f_ SW480 cells. (**G**) The levels of *SOX2* mRNA in control and *Ec*PDF_3f_ SW480 cells. (**H**) Immunoblotting of SOX2 in control and *Ec*PDF_3f_ SW480 cells with or without actinonin (2 µg/ml) treatment for 2 days. (**I**) Quantification of data in (**H**). **(J)** The growth of vector only-expressing (control) and *Ec*PDF_3f_-expressing HT29 cells. (**K**) Immunoblotting of SOX2 in control and *Ec*PDF_3f_-expressing HT29 cells. (**L**) Same as in (**F**) but with control and *Ec*PDF_3f_-expressing HT29 cells. In (**B**,**D**,**E**,**G**,**I**,**J**), error bars are presented as the mean ± SD of three replicate experiments; two-tailed *t*-test; significant *P* values are indicated in the figures.

In addition to promoting growth, *Ec*PDF_3f_ expression in SW480 cells induced notable morphological changes, including changes in cell shape, colonization pattern, and cell size (Fig. 2A). In particular, these cells exhibited a reduced size, one of properties of stem cells^13,14^ (Fig. 2B). Spheroid assays assessing the self-renewal capacity of cells demonstrated that the expression of *Ec*PDF_3f_ caused SW480 cells to form approximately five times more tumor-derived spheroids than the vector alone (Fig. 2C,D).

Moreover, quantitative reverse transcriptase-polymerase chain reaction (qRT-PCR) revealed strong upregulation of the mRNA levels of the cell surface glycoprotein CD24 upon *Ec*PDF_3f_ expression (Fig. 2E). Consistently, fluorescence-activated cell sorting (FACS) analyses demonstrated a higher percentage of CD24-positive cells in *Ec*PDF_3f_ cells compared with vector-control cells (Fig. 2F). (In this experimental setting, the observed decrease in the non-specific background signal of isotype control for CD24 in *Ec*PDF_3f_ SW480 cells, compared with that in vector only-control cells, likely stemmed from inherent cellular variations, such as differences in cell size.) This result is in agreement with a previous finding that a subpopulation of CD24-positive cells in colon cancer cell lines has CSC characteristics^15,16^.

Furthermore, *Ec*PDF_3f_ expression in SW480 increased the mRNA and protein levels of the stem cell transcription factor SOX2^16^ (Fig. 2G,H). *Ec*PDF_3f_-mediated upregulation of SOX2 was partially reversed by the PDF inhibitor, actinonin (Fig. 2H,I). Notably, the partial (incomplete) decrease in SOX2 levels in the presence of actinonin may result from either the suboptimal inhibition of *Ec*PDF by actinonin or the indirect upregulation of SOX2 levels by *Ec*PDF expression, independent of its enzymatic activity; this requires further investigation.

To explore the impact of cytosolic Nt-deformylation beyond SW480 cells, we investigated HT29 cells, another colorectal cancer line. *Ec*PDF_3f_ expression significantly increased HT29 cell growth, albeit to a lesser extent than in SW480 cells (Figs. 1E, 2J). Similarly, *Ec*PDF_3f_ discernably upregulated SOX2 expression in HT29 cells (Fig. 2K). However, unlike SW480 cells, *Ec*PDF_3f_ did not further elevate CD24 levels in HT29 cells, where CD24 expression was already high and likely saturated compared to that in SW480 cells (Fig. 2F,L). Given that HT29 cells express lower levels of Nt-formylated proteins than SW480 cells^9^, the effects of *Ec*PDF_3f_ expression on HT29 cells may be comparatively subdued. Overall, these data suggest that cytosolic Nt-deformylation can induces CSC-like properties in other colorectal cancer cells to varying extents.

### EcPDF expression enhances tumor growth in colorectal cancer xenografts

**Figure 3:**
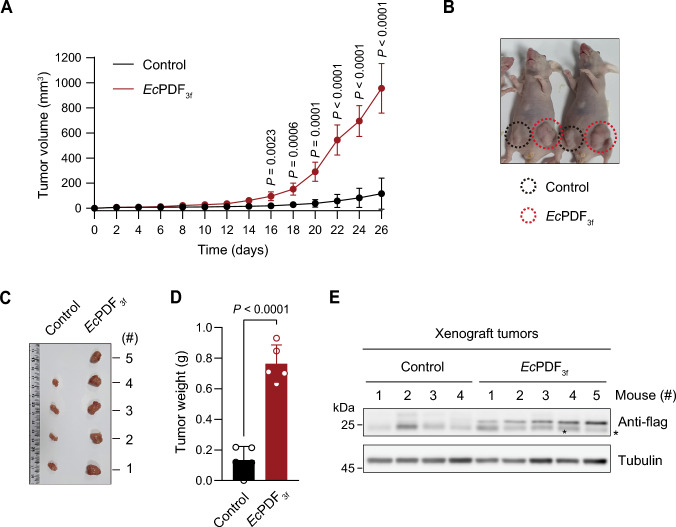
*Ec*PDF expression increases tumorigenicity in the in vivo xenograft model. (**A**) In vivo subcutaneous tumor growth curves of control and *Ec*PDF_3f_ SW480 cells. Tumor volumes were subsequently measured every 2 days until day 26. Data in the graph are presented as the mean ± SD of five mice per sample; two-tailed *t*-test; significant *P* values are indicated in the figure. (**B**) Representative image of xenograft mice on day 26 post-injection. (**C**) Images of the control and *Ec*PDF_3f_ SW480 xenograft tumors dissected from five different mice (#1–5) on day 26 post-injection. (**D**) The weights of the xenograft tumors in (**C**). Error bars are presented as the mean ± SD of five xenograft tumors per sample; two-tailed *t*-test; significant *P* value is indicated in the figure. (**E**) Same as in (**C**) but immunoblotting of *Ec*PDF_3f_.

We determined the tumorigenic potential of *Ec*PDF_3f_ SW480 colorectal cancer cells using in vivo xenograft models given the acquired CSC-like features and accelerated growth upon cytosolic deformylation of Nt-formylated proteins (Fig. 3A–D). Control (vector alone) and *Ec*PDF_3f_ SW480 cells were subcutaneously injected into the posterior flanks of nude mice and the tumor volume was measured every two days (Fig. 3A). The tumors of *Ec*PDF_3f_ SW480 cells grew faster and formed larger volumes and weights than those of control cells (Fig. 3A–D). Notably, *Ec*PDF_3f_ expression in *Ec*PDF_3f_ SW480 cells was maintained until the date of tumor extraction (Fig. 3E).

**Figure 4:**
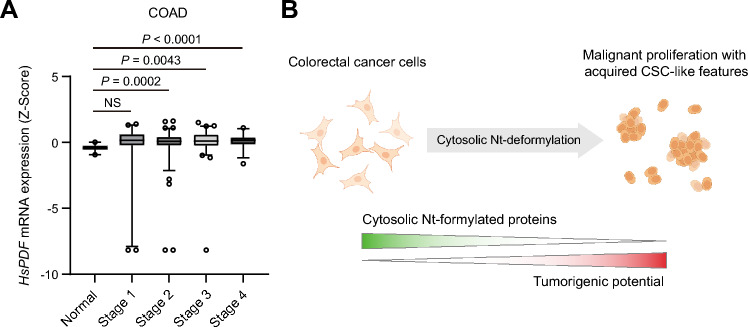
Proposed model for cytosolic deformylation as a critical driver of tumorigenesis. (**A**) Comparative TCGA data on the relative levels of *HsPDF* mRNA in normal tissues (*n* = 41) versus colorectal adenocarcinoma (COAD) tissues in various stages (*n* = 473). In the box-and-whisker plot, the boxes span the 25–75th percentiles with the median, while the whiskers extend to the 2.5–97.5th percentiles. Individual dots represent outliers. Two-tailed *t*-tests; significant *P* values are indicated in the figure; *NS* not significant. (**B**) A schematic depicting the effect of cytosolic Nt-deformylation on tumorigenicity of colorectal cancer cells. Downregulation of cytosolic Nt-formylated proteins promotes the tumorigenic potential of colorectal cancer cells by inducing CSC-like characteristics, such as reduced cell size, enhanced self-renewal capacity, and elevated expression of CSC marker proteins.

Human cells contain their own *Hs*PDF deformylase^17^. Interestingly, analysis of TCGA database revealed a substantial upregulation of *Hs*PDF mRNA levels in colorectal adenocarcinoma (COAD) patient tissues compared to normal tissues (Fig. 4A). This upregulation is further accentuated in the later stage of colorectal cancer progression (Fig. 4A). Consequently, endogenous *Hs*PDF-mediated deformylation of Nt-formylated proteins also partially and likely contributes to tumorigenesis in colorectal cancers.

In sum, these findings indicate that cytosolic Nt-deformylation enhances the tumorigenic potential by promoting the proliferative and CSC-like properties of colorectal cancer cells (Fig. 4B).

## Discussion

We explored the pathophysiological role of cytosolic Nt-formylation in human colorectal cancer cells by monitoring the effects of deformylation of Nt-formylated proteins (through ectopic expression of *Ec*PDF deformylase). The resulting deformylation dramatically increased cell proliferation, CSC-like phenotype, and in vivo xenograft tumorigenesis (Figs. 1, 2, 3). The reliance of cancer cell growth and tumorigenesis on the downregulation of cytosolic Nt-formylated proteins underscores the pathophysiological importance of naturally occurring cytosolic Nt-formylated proteins (peptides) in the suppression of cancer cell growth.

Endogenous *Hs*PDF deformylase is normally localized in the mitochondria^17^. Numerous mitochondria-targeting proteins are often detected in the cytosol^8,18,19^; therefore, functionally effective populations of *Hs*PDF may also be present in the cytosol to facilitate deformylation of cytosolic Nt-formylated proteins. Interestingly, *Hs*PDF was frequently upregulated in colorectal and other cancer tissues (Fig. 4A), making it a promising target for anti-cancer drugs^20^. *Hs*PDF inhibitors such as actinonin suppress the proliferation of various cancers^12,21^. While these drugs are presumed to exert their antitumorigenic activity by inhibiting mitochondrial *Hs*PDF, the present study suggests that inhibition of cytosol-retained *Hs*PDF could be one of the possible mechanisms underlying the action of PDF inhibitors.

Surprisingly, this study also revealed that the overall phenotypic characteristics of *Ec*PDF-expressing SW480 cells are analogous to those of stem cell transcription factor OCT4-expressing melanoma^22^ (Fig. 2). Morphological transformation, acquisition of CSC-like features, and increased tumorigenesis were similar between *Ec*PDF-expressing SW480 cells and OCT4-expressing melanoma cells (Oct4 reprograms adult stem cells to induced pluripotent stem [iPS] cells as a single factor)^23,24^ (Figs. 2, 3). Therefore, it would be interesting to explore strategies aimed at downregulating cytosolic Nt-formylated proteins, such as the ectopic expression of *Ec*PDF, in the induction of pluripotent stem cells or organ development.

In conclusion, this study demonstrated a negative correlation between cytosolic Nt-formylation and the malignant properties of SW480 colorectal cancer cells. Its potential as a previously unknown biomarker for cancer classification and treatment holds great promise, although further investigation is required to elucidate the precise effects of cytosolic Nt-formylation.

### Supplementary Information

Supplementary Information.

## Data Availability

All data are available in the main text and Supplementary Information. Further details can be obtained from the corresponding authors (C.-S.H and Y.L) on reasonable request.
