# Supplementary material for: Cytosolic N-terminal formyl-methionine deformylation derives cancer stem cell features and tumor progression
Source: Sci Rep. 2024 Jun 28;14:14900. doi: 10.1038/s41598-024-65701-1 (PMC11213908; doi:10.1038/s41598-024-65701-1)

Supplementary Information

Supplementary Tables 1–4.

Original Immunoblotting Images.

Supplementary Table 1 Plasmids used in this study

Plasmid	Description	Source
pcDNA3(+)	Mammalian expression vector with a CMV promoter	Lab collection
OGS411	Mammalian expression vector with dual CMV promoters	Lab collection
pCH5540	P_{CMV1}-*Ec*PDF_{3f} and P_{CMV2} in OGS411	Lab collection
pCH5542	P_{CMV}-*Ec*PDF_{3f} in pcDNA3(+)	This study

Supplementary Table 2 Primers for plasmids used in this study.

Primer	Sequence
OCH5576	5′-AATTCGACTACAAAGACCATGACGGTGATTATAAAGATCATGACATCGACTA-3′
OCH5577	5′-AATCTCGAGTCACTTGTCATCGTCATCCTTGTAGTCGATGTCATGATCTTTA-3′
OCH5578	5′-AATGGTACCATGTCAGTTTTGCAAGTG-3′
OCH5579	5′-ATGGTCTTTGTAGTCGAATTCAGCCCGGGCTTTCAGACG-3′

Supplementary Table 3 Human cell lines used in this study

Name	Description	Source	Catalog #
SW480	Human colorectal carcinoma	KCLB	10228
Vector	Vector-expressing stable SW480	This study	N/A
*Ec*PDF$_{3f}$-1	*Ec*PDF$_{3f}$-expressing stable SW480	This study	N/A
*Ec*PDF$_{3f}$-2	*Ec*PDF$_{3f}$-expressing stable SW480	This study	N/A
HT29	Human colorectal carcinoma	ATCC	HTB-38

Supplementary Table 4 RT-qPCR primers used in this study.

Gene	Forward	Reverse
SOX2	5′-CACAACTCGGAGATCAGCAA-3′	5′-CGGGGCCGGTATTTATAATC-3′
CD24	5′-CTGCTGGCACTGCTCCTAC-3′	5′-GGTGGTGGCATTAGTTGGAT-3′
β-actin	5′-GCCTCGCCTTTGCCGATCC-3′	5′-CCTTGCACATGCCGGAGCC-3′

Original Immunoblotting Images.

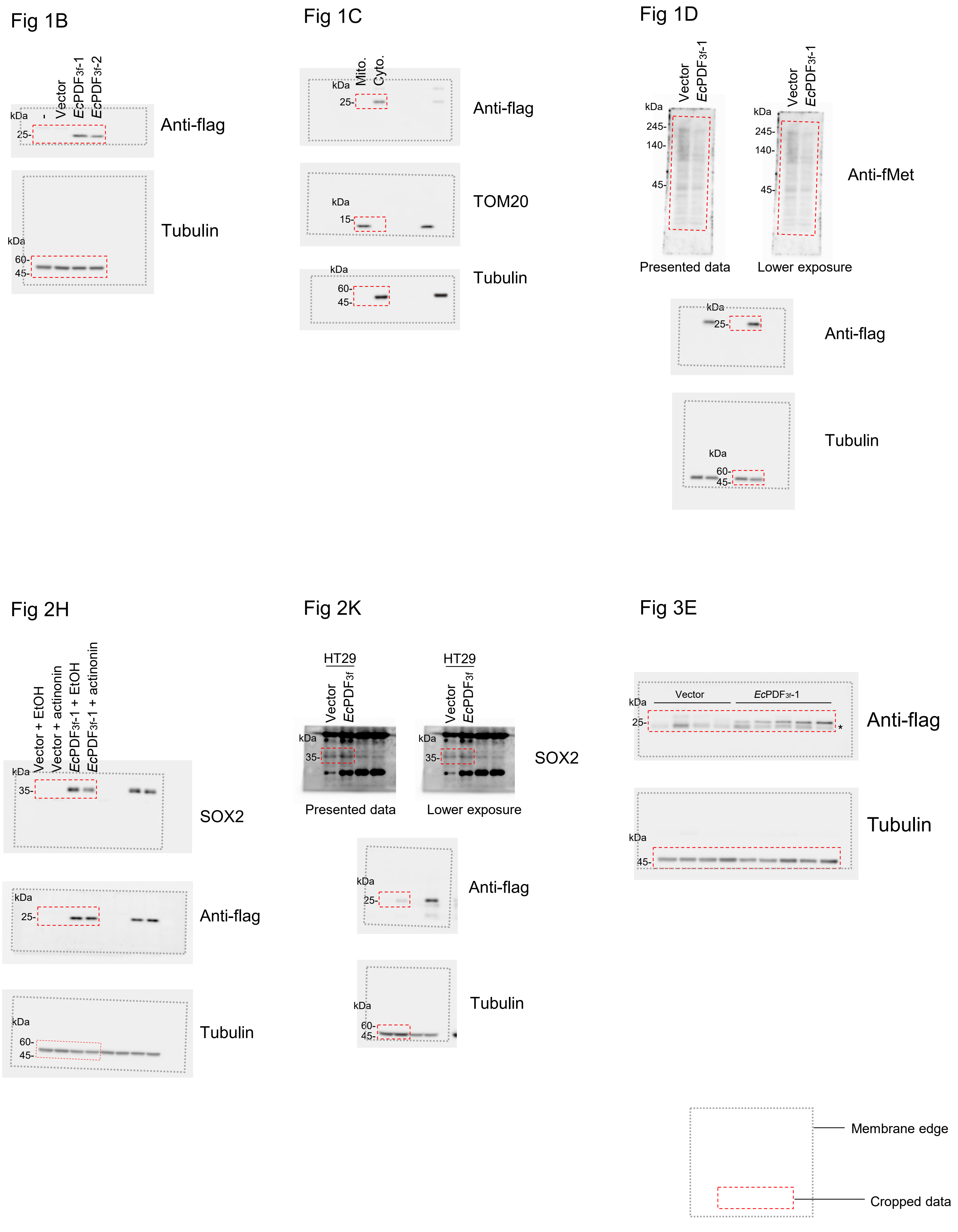

Supplement: Supplementary file 1 — Supplementary Information. [file 41598_2024_65701_MOESM1_ESM.pdf]
